# Diphtheria—Treatment of

**Published:** 1883-04-20

**Authors:** Addison C. Fox

**Affiliations:** Maryland


### Southern Medical Record:

EDITORS:

T. S. POWELL, M.D. W. T. GOLDSMITH, M.D. R. C. WORD, M.D.

*R. C. WORD, M.D., Managing Editor.*

☞All Communications and Letters on Business connected with the RECORD must be addressed to the Managing Editor.

VOL. XIII. ATLANTA, GA., APRIL 20, 1883. NO. 4.

### ORIGINAL AND SELECTED ARTICLES.

### DIPHTHERIA—TREATMENT OF.

BY ADDISON C. FOX, M. D., OF MARYLAND.

I deem it my professional duty to give to the public the following remedy for diphtheria. This remedy has not failed me, nor two other physicians to whom I gave the recipe, *in a single instance*, although since we commenced its use, about six months ago, we have treated a large number of cases—mild, malignant, and some complicated with croup and scarlet fever. Believing that the false membrane in diphtheria is alive with *bacteria*, and that even a weak solution of mercury or thymol would destroy them in a short time, I prepared the following gargle:

℞ Hydr. bichloridi........................... gr. j, (1)

Thymol (crystals)......................... gr. ss, (½)

Alcohol.................................. ℥ ii, (2)

Tr. phytolacca dec........................ ℥ ss, (½)

Aqua dest... .............................. ℥ xiv (14)

M. Sig. To be used freely as a gargle every one, two or three hours.

I have often seen the false membrane disappear in twenty-four hours, under this treatment, (before the bacteria could enter the blood) and seldom later than forty-eight hours. I give internally, for the blood poison, to clean the tongue and promote the proper absorption of food, simply the tincture of baptisia tinctoria in one drop doses in a teaspoonful of water, every hour or two. I also give freely a diet of milk, cream and beef essence, and milk toddy, if needed, and insist on free ventilation and the use of disinfectants.

When the child is too young to use the gargle, I make a mop by tightly tying absorbent cotton to a pen-handle or stick, or rolling it in the dry powder until covered, and then applying gently and carefully to the membrane in the throat, three or four times daily. The powder is prepared as follows :

℞ Hydrarg. bichloride .......................... gr. j,

Sacchari lactis.............................. ʒ i-ʒii,

Ol. menth. pip.............................. gtt. ii.

M. This powder must be applied *dry* with the *dry* mop, renewing the cotton each application.

I also send the following remedy for "Membranous Croup," which, in a practice of over twenty years, I have never seen, equalled :

℞ Pulv. sanguinaria rad.......................... ʒ j,

Acid acetic dilut.............................. ℥ viii,

Aqua destil.................................. ℥ viii.

Mix and let stand seven days, then filter, add two pounds white sugar and boil until the sugar is dissolved. Strain the syrup and to each ounce add tinc. aconite root and tinc. iodine of each one drop. Give one-half teaspoonful, to a child one year old, every half hour until relieved. This syrup is also useful in diphtheria.

